# Bone Mesenchymal Stem Cell-Derived Exosome-Enclosed miR-181a Induces CD4^+^CD25^+^FOXP3^+^ Regulatory T Cells via SIRT1/Acetylation-Mediated FOXP3 Stabilization

**DOI:** 10.1155/2022/8890434

**Published:** 2022-05-26

**Authors:** Renyong Wang, Ruixue Li, Tiehan Li, Lei Zhu, Zongze Qi, Xiaokui Yang, Huan Wang, Baoquan Cao, Hong Zhu

**Affiliations:** ^1^First Department of Hepatobiliary and Pancreatic Surgery, The Second Affiliated Hospital of Kunming Medical University, Kunming, China; ^2^Department of General Surgery, The First People's Hospital of Xundian Hui and Yi Autonomous County, Kunming, China

## Abstract

Bone marrow mesenchymal stem cells (BMSCs) have been identified as a potential therapeutic approach to immune-related diseases. Here, we show that BMSC-derived exosomes promote FOXP3 expression and induce the conversion of CD4^+^ T cells into CD4^+^CD25^+^FOXP3^+^ Treg cells, which is significant for immunosuppressive activity. We found that miR-181a-5p is upregulated in BMSC-derived exosomes and can be transferred to CD4^+^ T cells. In CD4^+^ cells, miR-181a directly targets SIRT1 and suppresses its expression. Moreover, downregulated SIRT1 enhances FOXP3 via protein acetylation. In conclusion, our data demonstrated that BMSC-derived exosomal miR-181a is critical in the maintenance of immune tolerance. Furthermore, our results reveal that BMSC-derived exosomal miR-181a induces the production of CD4^+^CD25^+^FOXP3^+^ Treg cells via SIRT1/acetylation/FOXP3.

## 1. Introduction

Pancreas transplantation is widely used for treating diabetes mellitus [1, 2]. However, recurrent autoimmunity and conventional allograft rejection are significant obstacles to pancreas transplantation [1, 2]. Learned tolerance is a hallmark of the immune system, and the induction of immune tolerance is considered a promising way to improve the success of pancreas transplantation [3, 4].

MSCs are multipotent stromal cells that play a significant role in the immune response via immune suppression [5]. Zhang et al. showed the role of MSC-mediated immunosuppression in immune thrombocytopenia [6]. Mounayar et al. suggested that PI3k*α* and STAT1 modulate immunosuppressive activity by MSCs [7]. Exosomes are a type of membrane microvesicles approximately 40–150 nm in diameter [8] that are involved in Treg cell development [8, 9] and can mediate cellular communication by carrying miRNAs to neighboring cells [10]. Research suggests that stem cell-derived exosomes could be a new strategy for the treatment of neurodegenerative diseases [11]. In addition, accumulating evidence indicates that MSC-derived exosomal miRNAs are critical for immunosuppression regulation. Du et al. showed that MSC-derived cells promote immunosuppression of regulatory T cells in asthma [12]. Shahir et al. indicated that MSC-derived exosomes could induce mouse tolerogenic dendritic cells [13]. Moreover, MSC-derived exosomal miRNAs function in immunosuppression [14]. MSC-derived exosomes can transfer microRNAs (miRNAs) to receptors, subsequently affecting immune homeostasis [15–17].

Moreover, previous studies found that MSCs seem to play a significant role in inducing FOXP3-expressing Treg cells [18, 19]. Forkhead box protein 3 (FOXP3)-expressing CD4^+^CD25^+^ Treg cells are critical for immune tolerance maintenance, for example, Nemo-like kinase-enhanced FOXP3 participates in Treg cell-mediated immune tolerance [20]. FOXP3^+^ Treg cells promote transplantation tolerance via neuropilin-1 [21]. POH1 contributes to immune tolerance by maintaining FOXP3^+^ Treg cells [22]. Increasing evidence suggests that the maintenance of FOXP3 expression is critical for Treg cell development and function. Jang et al. indicated that Hhex suppresses Treg cells by inhibiting FOXP3 [23]. Chen demonstrated that dysregulation of FOXP3 by hypermethylation impairs the function of Treg cells [24]. FOXP3 also plays a central role in immune tolerance; thus, stabilization of FOXP3 expression may provide an acceptable way to maintain immune tolerance and improve the success of pancreas transplantation [25]. Researchers have demonstrated that FOXP3 expression and activity could be controlled by posttranslational modifications. Moreover, posttranslational modifications of FOXP3 contribute to Treg cell function [26]. Kagoya et al. indicated that arginine methylation of FOXP3 plays a crucial role in the suppressive activity of Treg cells [27]. Lin et al. suggested that kaempferol promotes the suppressive function of Treg cells by inhibiting PIMI-mediated FOXP3 phosphorylation [28]. In addition, the deacetylation of FOXP3 by sirtuin 1 (SIRT1) also functions in Treg cell regulation [29–31]. It was reported that acetylation of FOXP3 modulates the suppressive function of CD4^+^CD25^+^ FOXP3^+^ Treg cells [29, 30]. Zhang et al. showed that miR-23a-3p-mediated FOXP3 acetylation could induce Treg function [32]. In abdominal aortic aneurysm (AAA), SIRT1-regulated acetylation of FOXP3 modulates Treg function [30]. Forkhead box protein 3 (FOXP3)-expressing CD4^+^CD25^+^ Treg cells play an essential role in immune tolerance maintenance [33]. Sustained FOXP3 expression is the most specific marker for characterizing CD4^+^CD25^+^FOXP3^+^ Treg cells [23, 34]. Therefore, the regulation of FOXP3 may provide a potential method for immunosuppression. Epigenetic regulation, such as acetylation and methylation, of FOXP3 has been well studied [35].

In this study, we uncovered the underlying mechanism by which BMSC-derived exosomal miR-181a induces CD4^+^CD25^+^FOXP3^+^ Treg cells via SIRT1/acetylation/FOXP3, providing a potential way to improve the success of pancreas transplantation.

## 2. Materials and Methods

### 2.1. Cell Culture

BMSCs were purchased from Cyagen Biosciences (MUBMX-01001). Then, the cells were cultured in Mouse Mesenchymal Stem Cell Growth Medium (MUCMX-90011, Cyagen Biosciences) and cultured at 37°C and 5% CO_2_. CD34 and CD44 surface markers were used for BMSC analysis.

### 2.2. BMSC-Exosome Isolation and Identification

When the density of BMSCs reached approximately 80%, the culture medium was discarded, and serum-free medium for BMSCs was added. After culturing for 24 h, the supernatant was aspirated into a 50 ml centrifuge tube and subjected to gradient centrifugation (300 g, 10 min; 2000 g, 10 min; 10000 g, 30 min) at 4°C. The supernatant was transferred to an exosome extraction ultracentrifuge tube and subjected to centrifugation (100000 g, 70 min). The supernatant was discarded, and the sediment was washed with PBS and subjected to centrifugation (100000 g, 70 min). The exosomes were resuspended in 150 *μ*l PBS and identified with transmission electron microscopy as described previously [36].

### 2.3. CD4^+^ T Cell Isolation and Purification

CD4^+^ T cells from the spleen were isolated using magnetic activated cell sorting (MACS). Briefly, a spleen cell suspension was obtained by grinding the tissue. After lysis, the cells were resuspended in PBE buffer. Anti-CD4 magnetic beads (Miltenyi) were used to isolate CD4^+^ T cells following the manufacturer's protocol.

### 2.4. Flow Cytometry

Flow cytometry analysis was performed to determine the percentage of Treg cells in CD4^+^ T cells. Treg cells were measured by flow cytometry with FOXP3^+^ as the marker. Briefly, the cells were first stained with anti-CD4-FITC (ab218745, Abcam), anti-CD25-PE (ab210334, Abcam), and anti-FOXP3-APC (ab200568, Abcam) antibodies. Fluorescence signals were measured by a FACS Fortessa system (BD).

### 2.5. Cell Transfection

Cells were transfected with miR-181a inhibitor (5′-ACUCACCGACAGCGUUGAAUGUU-3′) and miR-181a NC (5′-CAGUACUUUUGUGUAGUACAA-3′) using Lipofectamine 2000 reagent (Invitrogen) according to the manufacturer's instructions.

### 2.6. Reverse Transcription-Quantitative (RT-q) PCR Analysis

RT-qPCR was used to examine the expression of miR-181a. Total RNA was isolated using TRIzol reagent (R0016, Beyotime), and 1 *μ*g RNA was used as a template for cDNA synthesis using SuperScript III RT (18080093, Invitrogen). The primers used in this study were as follows: miR-181a-5p forward primer: 5′-CGGCAACATTCAACGCTGT-3′ and reverse primer: 5′-GTGCAGGGTCCGAGGTATTC-3′; U6 forward primer: 5′-CTTCGGCAGCACATATAC-3′ and reverse primer: 5′-GAACGCTTCACGAATTTGC-3′. RT-qPCR was performed at 95°C for 3 min, 95°C for 5 s, 56°C for 10 s, 75°C for 25 s (39 cycles), 65°C for 5 s, and 95°C for 50 s.

### 2.7. Western Blotting

Total proteins were extracted by RIPA lysis buffer (Beyotime, P0013B), and the concentration of the proteins was measured by a BCA kit (Beyotime, P0012). Equal amounts of protein lysates were loaded on a sodium dodecyl sulfonate-polyacrylamide gel (SDS–PAGE) and transferred to a polyvinylidene fluoride membrane. The membrane was blocked with 5% nonfat milk and incubated with antibodies at 4°C overnight. The primary antibodies used were as follows: anti-CD81 (1 : 1000, Cell Signaling Technology, 56039), anti-CD63 (1 : 1000, Abcam, ab68418), anti-CD9 (1 : 1000, Abcam, ab223052), anti-SIRT1 (1 : 1000, Abcam, ab263965), and anti-FOXP3 (1 : 2000, Abcam, ab10901). GAPDH was used as a loading control. Then, a horseradish peroxidase (HRP)-labeled secondary antibody was used to detect the specific protein bands.

### 2.8. Immunoprecipitation

The acetylation of FOXP3 was detected using an IP kit (Absin, abs955-50 tests) according to the manufacturer's instructions. Briefly, the collected cells were washed with PBS and lysed with IP lysis buffer on ice for 5 min. Cells were scraped from the plate and transferred to a microcentrifuge tube. After ultrasonic disruption 3 times, the cells were subjected to centrifugation (14,000 g, 10 min) at 4°C, and the supernatant (cell lysate) was transferred to a new tube. Cell lysates (200–1000 *µ*g total protein) were mixed with anti-FOXP3 antibody. After overnight incubation at 4°C, the protein A/G plus agarose was added to the sample and incubated on a rotator at 4°C for 2 hours. The mixture was centrifuged at 12,000 g for 1 minute to retain the precipitate, and it was washed with wash buffer. The acetylation of FOXP3 was determined by Western blotting with antiacetylated-lysine antibody (Cell Signaling Technology, 9941) and anti-FOXP3 antibody (Abcam, ab10901).

### 2.9. Luciferase Reporter Assay

The wild (WT) or mutant (MUT) type of the 3′-UTR of SIRT1 was inserted into the pGL3 promoter vector (Promega, E1761). SIRT1 WT or SIRT1 MUT and miR-181a control or miR-181a mimic were transfected into HEK-293T cells (Procell, CL-0005). The luciferase activities were measured by the Dual-Luciferase Reporter Assay System.

### 2.10. Statistical Analysis

All of the data are presented as the mean ± SD as indicated for at least three independent experiments and were tested with Student's *t*-test for between-group differences. *P* < 0.05 was considered statistically significant.

## 3. Results

### 3.1. Characterization of BMSCs and BMSC-Derived Exosomes

**Figure 1:**
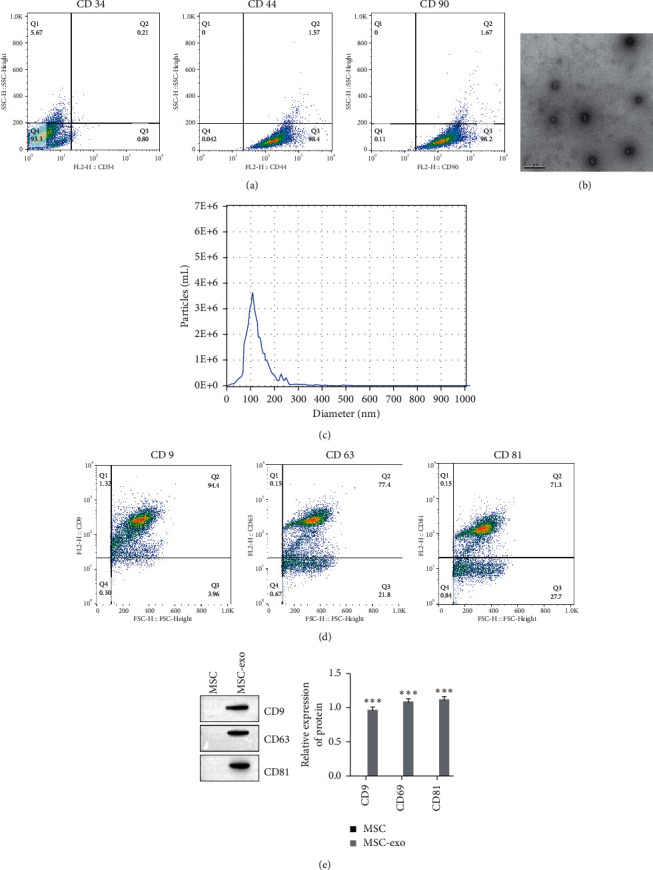
Characterization of BMSC and BMSC-derived exosomes. (a) CD34, CD44, and CD90 surface markers of the cells measured by flow cytometry. (b) Exosomes isolated from BMSCs detected by transmission electron microscopy. (c) Exosome diameter measured by dynamic light scattering (DLS). (d) Expression of exosome markers detected by flow cytometry. (e) Expression of exosome markers detected by Western blot.

We first identified BMSCs by detecting the CD34 and CD44 surface markers of the cells (Figure 1(a)). Exosomes derived from MSCs were identified with transmission electron microscopy (Figure 1(b)). Western blotting results indicated that the exosome markers CD9, CD63, and CD81 in the exosomes were significantly higher than those in the BMSC lysate (Figure 1(c)).

### 3.2. miR-181a Is Highly Expressed in BMSC-Derived Exosomes

**Figure 2:**
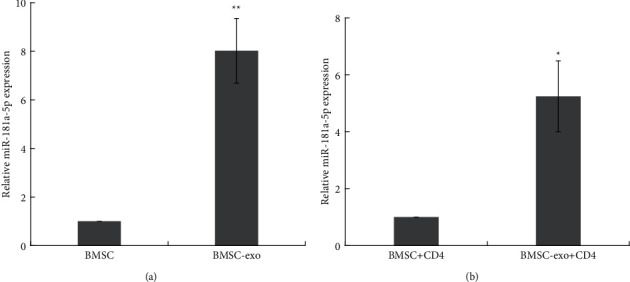
miR-181a is highly expressed in BMSC-derived exosomes. (a) Expression of miR-181a in BMSC-derived exosomes measured by RT-qPCR assay. ^*∗∗*^*P* < 0.01. (b) Expression of miR-181a in CD4^+^ T cells treated with BMSC-derived exosomes measured by RT-qPCR assay. ^*∗*^*P* < 0.05. Data are the mean ± SD (*n* = 3 biological replicates).

To determine the expression of miR-181a in the BMSC-derived exosomes, we first performed RT-qPCR to detect miR-181a expression in the BMSC-derived exosomes and BMSC lysates. As shown in Figure 2(a), the expression of miR-181a was upregulated in the BMSC-derived exosomes. In addition, after coculturing with the BMSC-derived exosomes, miR-181a expression was increased in the CD4^+^ cells (Figure 2(b)).

### 3.3. BMSC-Derived Exosome miR-181a Treatment Triggers the Conversion of Effector T Cells into FOXP3^+^ Expressing Tregs

**Figure 3:**
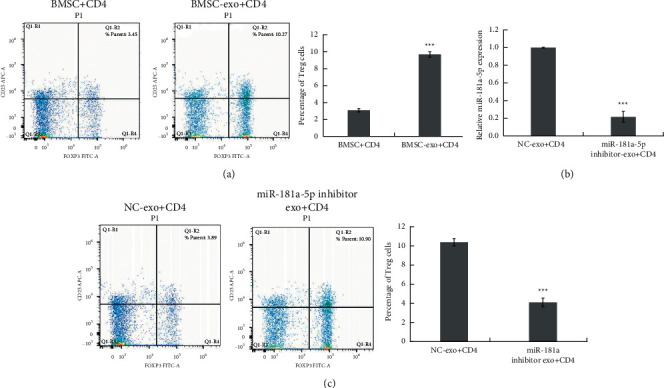
BMSC-derived exosome miR-181a treatment triggers the conversion of effector T cells into FOXP3^+^-expressing Tregs. (a) The frequency of CD4^+^CD25^+^FOXP3^+^ Treg cells in CD4^+^ T cells cocultured with BMSC-derived exosomes analyzed by flow cytometry. ^*∗∗∗*^*P* < 0.001. (b) miR-181a expression in CD4^+^ T cells treated with NC or miR-181a inhibitor-transfected BMSC-derived exosomes measured by RT-qPCR assay. ^*∗∗∗*^*P* < 0.001. (c) The frequency of CD4^+^CD25^+^FOXP3^+^ Treg cells in CD4^+^ T cells treated with NC or miR-181a inhibitor-transfected BMSC-derived exosomes analyzed by flow cytometry. ^*∗∗∗*^*P* < 0.001. Data are the mean ± SD (*n* = 3 biological replicates).

We next determined the role of MSC-derived exosome miR-181a (BMSC-exo-miR-181a) in the stimulation of CD4^+^CD25^+^FOXP3^+^ Treg cells. CD4^+^ cells were treated with BMSC-exo-miR-181a, and the frequency of CD4^+^CD25^+^FOXP3^+^ Treg cells was analyzed by flow cytometry. As shown in Figure 3(a), the frequency of CD4^+^CD25^+^FOXP3^+^ Treg cells in the BMSC-exo-miR-181a treated group was higher than that in the BMSC lysate treated group.

We next knocked down miR-181a by miR-181a inhibitor transfection into BMSCs and isolated exosomes from the knockdown BMSCs. The expression of miR-181a in exosomes derived from miR-181a inhibitor-transfected BMSCs was measured (Figure 3(b)). After inhibition of miR-181a, the exosomes no longer increased the frequency of CD4^+^CD25^+^FOXP3^+^ Treg cells (Figure 3(c)). These results revealed the function of BMSC-exo-miR-181a in maintaining CD4^+^CD25^+^FOXP3^+^ Treg cells.

### 3.4. BMSC-Derived Exosomal miR-181a Regulates FOXP3 via SIRT1-Mediated Acetylation

**Figure 4:**
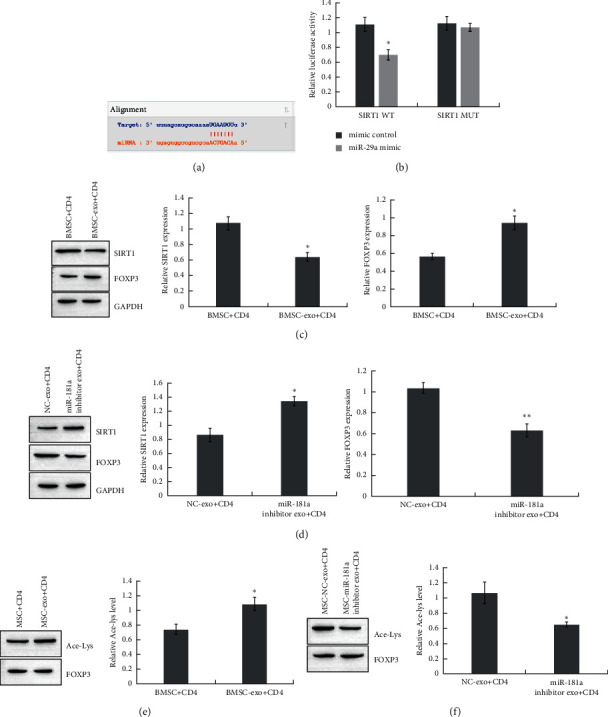
BMSC-derived exosomal miR-181a regulates FOXP3 via SIRT1-mediated acetylation. (a) The binding site of miR-181a and SIRT1 predicted by StarBase. (b) The interaction of SIRT1 and miR-181a determined by a dual-luciferase reporter assay. ^*∗*^*P* < 0.05. (c) Expression of SIRT1 and FOXP3 in CD4^+^ T cells treated with BMSC-derived exosomes detected by Western blot. ^*∗*^*P* < 0.05. (d) Expression of SIRT1 and FOXP3 in CD4^+^ T cells transfected with NC or miR-181a inhibitor BMSC-derived exosomes detected by Western blot. ^*∗*^*P* < 0.05. (e) FOXP3 acetylation in CD4^+^ T cells treated with BMSC-derived exosomes detected by immunoprecipitation. ^*∗*^*P* < 0.05. (f) FOXP3 acetylation in CD4^+^ T cells with CD4^+^ T cells transfected with NC or miR-181a inhibitor and BMSC-derived exosomes detected by immunoprecipitation. ^*∗*^*P* < 0.05. Data are the mean ± SD (*n* = 3 biological replicates).

miRNAs were previously reported to modulate target genes by binding to their 3′UTRs. Based on bioinformatics analysis, miR-181a could directly target a deacetylase, SIRT1 (Figure 4(a)). According to the dual-luciferase reporter assay, there was a relationship between miR-181a and SIRT1 (Figure 4(b)). After BMSC-exo-miR-181a treatment, the expression of SIRT1 in CD4^+^ cells decreased (Figure 4(c)). Inhibition of miR-181a rescued SIRT1 expression (Figure 4(d)).

Accumulating evidence has demonstrated that SIRT1 modulates FOXP3 expression via protein deacetylation. Here, we detected FOXP3 and acetylation levels in CD4^+^ cells treated with BMSC-exo-miR-181a. BMSC-exo-miR-181a promoted FOXP3 and acetylation (Figures 4(c) and 4(e)). The suppression of miR-181a decreased FOXP3 and acetylation levels (Figures 4(d) and 4(f)).

## 4. Discussion

Here, we demonstrate the effect of exosomes derived from bone marrow mesenchymal stem cells (BMSCs) on immunosuppressive regulation. Our results indicate that BMSC-derived exosomes can induce the transformation of CD4^+^ T cells into CD4^+^CD25^+^FOXP3^+^ Treg cells. CD4^+^CD25^+^FOXP3^+^Treg cells play a key role in the aggressiveness of diseases and cancers by regulating the immune response. In recent years, with advances in research, the regulatory mechanism of CD4^+^CD25^+^Foxp3^+^Treg cells in the process of controlling autoimmunity and maintaining immune tolerance has been gradually understood [37, 38].

In our study, it was demonstrated that miR-181A was highly expressed in BMSC-derived exosomes, and miR-181A, miR-181b, miR-181c, and miR-181D jointly formed the miR-181 family, which is one of the most abundant miRNAs in lymphatic tissues [39]. mir-181a plays an important role in B cell development in bone marrow [40, 41] and immune function [42]. We found that miR-181A can be internalized by CD4^+^ cells and that miR-181A in CD4^+^ cells directly target SIRT1. SIRT1 is a protein deacetylase that regulates protein expression through deacetylation. miRNA and host cell protein expression are important regulatory mechanisms. Studies have shown that HCV impairs the T cell response through miR-181a-mediated DUSP6 expression [43]. miR-181A not only regulates T cell response-related proteins but also balances immune-mediated virus clearance with inflammatory damage and enhances immune tolerance [44]. This study demonstrated that miR-181A has a targeted relationship with SIRT1, a deacetylase that regulates protein expression. In future studies, the proteins related to miR-181A that have roles in the process of immune tolerance can be further studied, and the related mechanisms can be explored. Our results suggest that the suppression of SIRT1 enhances FOXP3 activity by increasing acetylation levels. In addition, our results suggest that BMSC-derived exosomes trigger CD4^+^CD25^+^FOXP3^+^Treg cells through mir-181A/SIRT1-mediated FOXP3 acetylation.

Collectively, our data show that exosomes from bone marrow mesenchymal stem cells (BMSCs) induce the transformation of CD4^+^ T cells into CD4^+^CD25^+^FOXP3^+^ Treg cells. miR-181a is preferentially expressed in exosomes derived from bone marrow mesenchymal stem cells and can be transferred to CD4^+^ T cells. miR-181a directly targets SIRT1 in CD4^+^ T cells and reduces SIRT1 expression. Inhibition of SIRT1 enhances FOXP3 expression by promoting acetylation of FOXP3. We found that bmSC-derived exosomes carrying miR-181A induced the production of CD4^+^CD25^+^FOXP3^+^ Treg cells by regulating FOXP3 expression. In addition, we revealed the mechanism by which exosomal miR-181A enhances FOXP3 expression through sirT1-catalyzed acetylation. A limitation of this study is that we did not verify this mechanism in vivo.

## Data Availability

The data used to support the findings of this study are included within the article.
